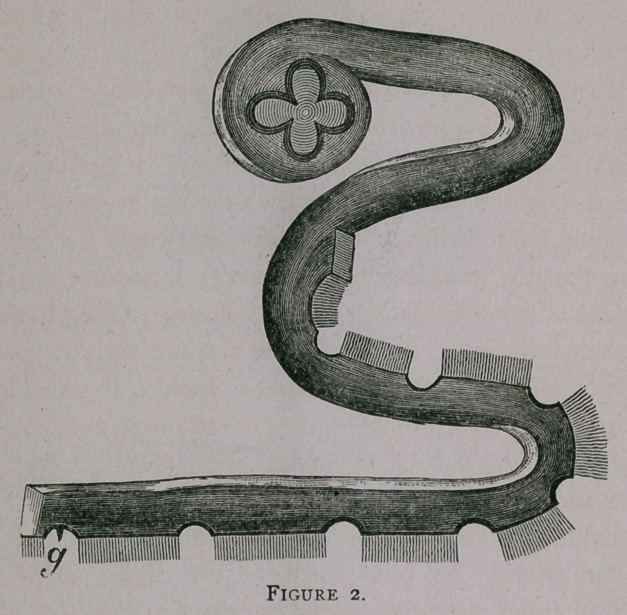# Energy of Nerve and Brain*Read before Alumni of Buffalo Medical University, February, 1884.

**Published:** 1885-04

**Authors:** W. H. Pitt


### ENERGY OF NERVE AND BRAIN.*

BY W. H. PITT, A. M., M. D.

In selecting nervous energy as the subject for a short paper, which I have the honor to present this evening, I did so because there is probably less known of the action of the nervous system than of any other organs of animal life; and what positive knowledge is possessed, we owe chiefly to the present generation, which has been so prolific in new discoveries in every department of science. Histology has grown with the microscope; embryology, with evolution; and physiology, with every advance in physics and chemistry. A degree of stability is now being realized in every branch of science which relates to the living, because the speculations concerning Nature in Aristotelian philosophy, have passed away.

And so, now, we have many of the phenomena relating to organic or inorganic matter accounted for and satisfactorily explained, since the general acceptance of the correlation of force and the conservation of energy.

It is understood that force or energy is something in motion or position in reference to some other thing. Conformable to this view, it should be borne in mind that the energies of the living being form no exceptions to the general rule or law. We speak of the radiant energy of the sun, and call it light and heat; of magnetic energy, of electric energy, of nervous energy, of

* Read before Alumni of Buffalo Medical University, February, 1884.

muscular energy; also, not least, of physical and chemical energy. We used to be told also of "vital energy"—a term which, however, lacks definition, and is becoming obsolete.

It is pleasant to find out, although somewhat late in the history of man, that all of the above energies are correlated, and that they are one and the same thing. I say thing, as they are merely matter in motion or position. Nervous energy is, therefore matter in motion, or in position to produce motion. All living beings have motion, and most of them nerves and nervous ganglia. Now there was a time when no life existed upon the earth; its temperature was too high to sustain life. When, however, its temperature fell to the proper point, and all other conditions were favorable, in the appointed time and manner, l:fe began. According to the accepted idea which so generally obtains, that beginning was very low in the scale of being; so low, indeed, that the organism was apparently of the same material throughout, albuminous, and commonly of a viscid fluid state. That this early form of life started from the hot rocks, there is every reason to believe. In North New Zealand, on the shores of Retnea, situated amidst boiling springs, a greenish, gelatinous, or slimy vegetable substance grows in the crevices of the rocks where the boiling spray constantly falls. In the Geyser regions of North America the protoplasmic vegetable matter grows on the hot rocks. But in the animal form, or among the rhizopods of primeval times, this semi-fluid state of existence must have been very unstable. If an individual happened to run against his neighbor, neither one was left to tell the story, as they coalesced like two drops of water. But in union there is strength, so this dual compound broke up into numerous particles, each one of which became a living creature. Such organisms are all stomach, as particles of food sink into them anywhere, and so they have no need of a mouth. They are likewise, all lung, respiratory at ever/ point breathing in oxygen, and if put into a vacuum will soon die. These living albuminous particles, were first observed under the microscope and noticed to possess mobility, and also that the movements did not obey the law of common fluid matter of that consistency. That there was some force or energy existing in the substance, was quite apparent, as it moved in directions contrary to gravity, and was both contractile and expansive at the same temperature. It was first discovered by Dujardine, a French naturalist about forty-six years ago, and called by him sarcode, in animal organisms of a very low order. Hugo Von Mohl found a similar substance in the cells of plants and named it *protoplasm.* Max Schultze perpetuated his name forever by demonstrating that the sarcode of the animal and the protoplasm of the plant were identical, and biological science took a long step forward. It now claims that this substance underlies all life, whether animal or vegetable, and has been appropriately called, by that great naturalist, Huxley, "the physical basis of life." You watch it under the microscope for hours, as it throws out its little arms (pseudopodia) and withdraws them, undulations pass over and through it, and it moves with considerable force. It is certainly the lowest form of life studied, as it is apparently of the same nature throughout as a living being, undifferentiated, "an organism without organs." Somewhat higher in the scale of beings is that little creature called *amœba*, which may be found in fresh water ponds, or in the gutters on the roofs of our houses. It is a marvelous point of living matter, a thousandth of an inch, or so, in diameter, and which, for a hundred years or more, has attracted the attention of microscopists and naturalists who are now only just beginning to comprehend its importance and its meaning. It moves over the field of view like the other protoplasm by improvising pseudopodia, but differs from its ancestors in having a nucleus, and within this nucleus a little point called the "contractile vacuole," which dilates and contracts incessantly. This throbbing point, embedded in the soft protoplasmic body is the first intimation of an organ supplying energy by its motion to the living creature,

In the transparent cells of the plant characæ, which grow in clear water streams, the protoplasm may be seen in motion, even with a low power microscope; and also in many other plants, particularly in the leaf cells of valimeria spiralis.

Here are some photographs of microscopic objects, greatly enlarged, taken by the Surgeon-General of the army, Washington, D. C., in which you see the corpuscles of the blood of the animals named on the plate are said to have amœba-like motion. Indeed, it is frequently noticed that the white corpuscles of human blood move across the glass under the microscope like an amœba, and some observers declare that they have been seen to devour their smaller companions, the red ones, which are supposed to supply them with oxygen. And so it appears that whether we examine plant or animal cell, which is common to both, we shall find it in some stage or other of life, furnished with living protoplasm.

All the phenomena of animal or vegetable life become better understood and more easily accounted for, by admitting the demonstrable fact that the cells are protoplasmic, having the property of contraction and expansion, and consequently power of receiving and imparting energy.

But the unit-like cell, amœba, dies as we have seen in a vacuum, mostly, from the exclusion of oxygen, but atmospheric pressure may have something to do with it. All things living, so far as known, labor under this pressure, and cannot thrive without its influence. It is about fifteen tons on a common-sized man. It must be very little on the amœba, still it is something, about twenty-four hundred thousandths of an ounce. So far as pressure, there is scarcely a limit to the amount which can be endured, providing that it pervades the whole organism, and is equal in all directions. And so considered in this sense the pressure by the amœba substance outward is just balanced by the surrounding pressure, leaving a free and easy movement to its projections, and the rythmic pulsation of its vacuole. A disturbance of the atmosphere, as waves passing through it, will produce undulations on the surface of the amœba, which proves that even this point of microscopic, living matter, receives and quickly transmits the energy imparted to it by the oscillating air. Vibrations passing through the medium in which the animal is bathed give up motion or energy, which may be retained for a time by the living thing or rapidly lost. If I jar a glass of water, undulations pass through it on account of its high elasticity, which, upon its surface, are called wavelets, and which you may also see pictured upon the ceiling if a light be held under the transparent glass. Now, anything alive in the water, a fish, for instance, would receive a part of this motion, and, if it has an inelastic apparatus, such as the brain and ganglia, for example, specially designed to stop this force, or energy, it might be stored up for future use, and expended in the performance of its own work and motions. If the water be illuminated or warmed it appears undisturbed so far as the human eye can detect; but there are, nevertheless, wonderfully small vibrations passing into it, which the fish may likewise, converting into its own variety, receive and appropriate.

What is true, in this respect, of the million-celled vertebrate fish, with its highly organized functions, in a quantity of water, is also true of the unicellular monera, or amœba, in a drop of water under the microscope.

As the physicist recognizes the same law governing the molecule and the planet, so the biologist accepts the microscopic, living protoplasm as the morphological unit, the primordial cell with which nature has built up, through wonderful modifications, all the myriads of complex and differentiated forms in the whole domain of life.

In this connection it should be borne in mind that all processes of Nature, whether taking place in animate or inanimate matter, depend upon that medium from which there is no escape and to which, probably, all phenomena of organisms should be referred.

Its vibrations are known to us as light, heat, electricity, magnetism, etc. As we know about the distance of the sun, the velocity of light has been determined.

There is a method, also, of arriving at the comparative pressure of two media, when the velocity of the vibrations through them is known, simply by a comparison of the square roots of their elasticities.

The velocity of sound in the air, deducting for the heat of progression, is 916.3 feet per second, while that of light through the ether is (using the greater number) 192,000 miles per second. The ratio is about 1,106,360 to 1. The pressure of the ether, therefore, is the square of this number times the atmospheric pressure, or eighteen and one-third billions of pounds per square inch, more than nine millions of tons. Enormous as this pressure appears, which is about the weight of a square mile of granite, still, as already shown, a living organism would be balanced in it, and so delicate a structure as our plastic amœba, which, according to these calculations, bears a weight of 2,750 pounds, would be able to move in any direction with the utmost ease. An absolutely elastic ether of this nature pervading all space, and filled with radiant force, would give up energy to all ponderable matter, whether living or dead. The *vis viva* of inert and the potential energy of animate matter, reappear as modified ethereal force. The earth receives its astounding share and transmits it in turn to all that move or exist upon its surface. The steel magnet draws its electricity from the magnetic earth, and keeps always full or saturated; notwithstanding, it may part with any quantity of magnetic energy to other steel bars, which, like itself, can impart to others without loss. Are all things alive bathed in the same ether and in contact with the same earth an exception? Do they, too, with specialized functions, derive energy from the same source and through the same media? Or, shall we say, because alive, they only accumulate energy from the food digested and the oxygen of the air? Let us look at our amœba once more. Its little arms are thrust out and withdrawn, its vacuole knows no rest. Can this bit of restless albumen get all its motion from molecular changes within itself in which we have discovered oxygen takes so prominent a part? Or, is the radiant energy of the sun a never-ending source of supply? Let us look for an answer to some of the lowest forms of life having anything like a nervous system. In the sea anemones there is no very definite nervous system. In the skin, however, and in the digestive tract, there are, according to the Hertwigs, modified epithelium cells with fine, hair-like threads, which perform the function of nerves. They also describe cells similar to the multipolar ganglion cells of the vertebrate which are sense cells in a transition state. The conclusion is that the nervous system has been derived from the superficial epithelium layer of the body. The special organs of sense or nerves, according to Balfour, are developed from the epi-blast of the ovum, and even the brain itself, as so graphically described by Huxley, is an infolding of the skin. And so it appears that the whole nervous system, brain and all, were developed from the general sense of feeling in the skin. Hearing, seeing, tasting, smelling, are modified senses derived from that of touch or feeling, and all of them supplied with nerves which act as conductors of force, as we shall see, to and from the ganglia and brain.

Before the remarkable researches of George J. Romanes, which were published in the *Fortnightly Review*, five or six years ago, it was a vexed question among biologists whether or not the *Medusa*, jelly-fish, had a definite nervous system, so difficult had the problem proved on account of the deliquescence of its tissues in the hands of the histologists. Romanes took hold of the subject physiologically and settled the question forever. As some of his rich discoveries bear directly upon the particular function of the nervous system which I wish to emphasize, your attention is directed to two or three views of the Medusæ on the chart before you, which were enlarged from his paper. Here is a variety called *aurelia aurita*, with its large swimming-bell, and pendent stomach and mouth, called the *polypite* (fig. 1). The

FIGURE 1.

ventral surface of the dome is concave, and consists, as also the surface of the polypite, of a neuro-muscular coat about the thickness of common paper. The dorsal integument is likewise very thin, and between the two is nothing but a mass of jelly. The jelly fish swims by contracting and expanding the lower coat. As the swimming-bell contracts, the water is forced out from the concavity behind, and by its reaction the animal is propelled. This expansion and contraction is constant and rythmical as the beating of the heart in a vertebrate animal. You will observe that the border of the bell is fringed with hair-like tentacles, and eight little spots, marginal bodies or ganglia. Romanes found that by cutting off this fringe all the way around the creature was paralyzed forever and sank helpless to the bottom; but that the fringe kept on pulsating for hours or even days. The swimming-bell, thus mutilated and apparently lifeless, was found, however, not to be dead, as it readily responded to artificial stimuli, contracting and expanding as before, but had no power of itself to move. He then varied the experiment by cutting out first one and then another of the marginal bodies or ganglia; but not till the last was removed did total paralysis ensue. It was, therefore, evident that these parts presided over muscular action. The next plate (fig. 2) is a view of the Medusa cut

FIGURE 2.

into a parallelogram, with the polypite section at one end and a single ganglion (*g*) at the other end, while the tentacles extend the length of one border of the ribbon-like strip. Now, if the opposite end be gently irritated with a camel's hair brush, a wave will pass to the polypite end and the ganglion will discharge reflexively, sending a pulse in a contrary direction. The tentacles being even more sensitive to stimulus than either the muscles or nerves, contract as the wave progresses in either direction, so that the velocity of the movement can be seen with the eye and its time noted. But it was with another species of Medusa which he experimented upon with light that he got the most marvelous effect. By subjecting it to a flood of intense light he discovered that the ganglia had the property of accumulating this energy, and then discharging it into the muscles of the swimming-bell, which produced mechanical motions as above described. Flashes of sunlight had the same effect, but the sumation or accumulation of the energy in the latter case took about one second of time before the reaction occurred. Curious to learn, poisons on the muscles and nerves of this low order of life, as his experiments directly prove, have the same effect, producing spasms, paroxysms, paralysis and death, as in the higher animals. As the jelly-fish is the lowest organism discovered, or perhaps that ever will be discovered, in which nerves or muscles make their first appearance, these brilliant discoveries, especially the one demonstrating that the ganglia store up energy for future use, make an important advance in knowledge, as relating to the physiology of the nervous system. But important as they are, particularly the one which alludes to the sumation of energy, the distinguished investigator seems not to have entertained the idea which naturally grows out of it, in its application to the nervous ganglia and brain of higher animal life.

I conclude, therefore, that the motion of man, that all the muscular energy he puts forth, in the shape of work performed, or lying potential, stored up in brain, gangalia, nerve and muscle, does not necessarily come from the chemical or mechanical change taking place in the food digested and assimilated; but that much of it does come from his environment, unbidden, the same way precisely, as we have seen, it may be accumulated by the Medusæ. According to the best authorities the average human adult develops, in twenty-four hours, and loses by radiation and otherwise, about 8,700 units of heat, equivalent to 3,000 foot-tons. The consumption of oxygen is one and one-fourth pounds ; the water nine ounces as a product of burnt hydrogen, and the carbonic dioxide gas nearly two pounds. The left ventrical does a work equal to ninety foot-tons, and the whole heart, about one hundred and twenty foot-tons. The urea eliminated is not far from 400 grams. The oxidation of the protein bodies .as measured by the amount of urea is about the same every day with very little increase by fatiguing labor. The expenditure of the food force and the inhaled oxygen in supplying the natural waste, keeping up motion, and the extravagant demands of radiation, would leave but a small fraction of energy to be expended by the common laborer in foot pounds as work. Indeed, careful estimates made on the quantity of albuminoids oxidized to water, carbonic dioxide and urea, in a man ascending a mountain, do not furnish half the energy necessary to lift his body that vertical hight. This was shown by Fick and Wislicenus in their ascent of the Faulhorn in the Alps, and they made no allowance for the force expended in radiation, or that exerted in muscular work voluntary or involuntary, (lungs, stomach, heart, etc). The fuel of muscular force is therefore not in the albuminous or nitrogenous portion, but must be found, if at all, to account for the work done in the non-nitrogenous compounds, such as the fats, sugars, glicogen, etc. But careful estimates of these. making full allowance for the force wasted in so many ways carrying on the functions of life, leave a large amount of energy still lacking to equal the work a strong healthy man is capable of performing in ten hours. The natural recourse left, from which this extra supply may be derived would seem to indicate a source external to the muscular animal. As the sun is the origin of all energy derived directly or indirectly from the earth and its atmosphere, some of its undulations, under whatever name, would keep the inelastic nerve centers saturated with force, which in turn would supply the protoplasmic muscular cells, producing tension, contractility and all the phenomena attendant upon mechanical motion. It is only upon some such view as this, I think, that physiological science can reach a satisfactory explanation of this intricate problem.

## Figures and Tables

**Figure 1. f1:**
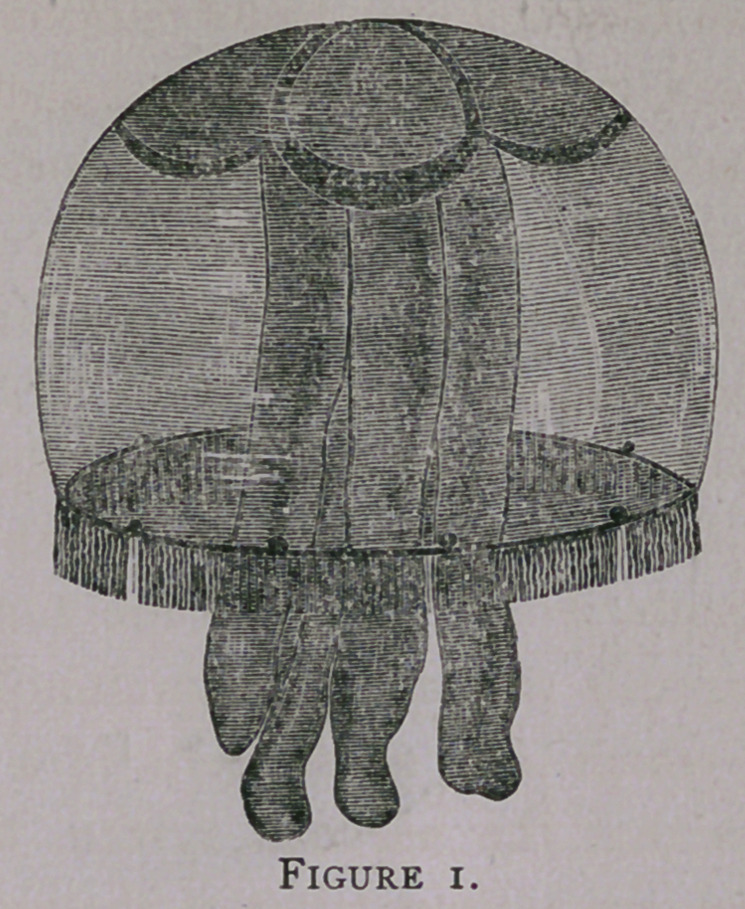


**Figure 2. f2:**